# Annotations

**Published:** 1909-08-07

**Authors:** 


### Annotations.

### A Legend with a Moral.

Among a large number of legends of the Cameroons, dealing quaintly with matters medical and non-medical, collected by M. Mansfeld and recently published in *Le Journal Médical Français*, there is one which explains the reason why the young of animals can walk as soon as they are born, whereas to the human infant this privilege has been denied. The account, which we have ventured to translate somewhat freely, runs as follows: " God created both men and goats; but to the first woman he gave a knife wherewith to sever the umbilical cord of her new-born infant. When the first kid was born into the world, his father, the buck, journeyed forth to seek the woman, and when he had found her he asked her to lend him the knife. But she refused, saying that the knife was meant only for the children of man. The buck thereupon complained to his Maker, who answered: ' The goats are in the right; I sent the knife for all. For his presumption and selfishness man will be punished. Henceforth the young of animals shall walk from the hour of birth, but the young of man shall only begin to walk at the end of a year . . . ' " Like many other such legends and fables, this seems to prove that our earliest ancestors were somewhat lacking in the higher moral qualities, especially in their dealings with the brute creation. But whereas they (and their descendants) were punished by appropriate physical misfortunes or disabilities, the experimental physiologist of to-day is visited with questions in Parliament, and brown dogs, and abusive epithets, and sometimes—at the dawn of the silly season—with rival " world congresses," each culminating in a triumphant gaily bannered mob-meeting in Hyde Park or Trafalgar Square.

### Nature and Nurture.

As recently hinted in these columns, for the meantime the subject of eugenics has had about enough ventilation. Unfortunately, the man in the street wants plenty of practical deductions, and in this field there are as yet no data on which to found them. Indeed, the whole *Grenzgebiet* of medicine and heredity requires not discussion, but serious, toilsome, new investigation. Probably the most important passage in the recently issued report of the deliberations of the Royal Society of Medicine on this subject was Dr. Bulloch's short speech to this effect. Nevertheless, popularisers of the cause of Nature rather than nurture have missed one point, namely, the artificiality of the present time and the delusions consequent thereon. We live in an age of baby foods, culture fads, and nostrums of every description—to touch only on the physical side. The possessor of conspicuous natural bodily aptitude has only to patent some device and proclaim it loudly enough as the secret of success in order to make a fortune. This is divertingly shown in the case of one of the many existing " physical culturists "—persons whose claims are smiled at by physiologists such as those who lately reformed physical exercise in the Army. At the beginning of this gentleman's career he was known to boast to an interviewer of exceptional powers from an early age, while later on, when commercial developments had supervened, it was system and régime, on the contrary, which had rescued him from semi-invalidism. Probably if one of the ladies whose shapely proportions (and lack of higher qualifications) are characteristic of the present musical comedy stage—if one of these ladies had cynical enterprise enough to " boom " energetically an ointment for developing the female figure, results would be highly remunerative too. In this country a national passion for athletics provides another rich field for similar exploitation. The man who " could play with a broomstick," if he is pecuniarily interested and worldly wise, obtrudes some mannerism of equipment on the public notice, and finds plenty of dupes to pay for it in expectation of success similar to his own. In fine, what this highly urbanised generation needs teaching is the truth of the old farmer's maxim, " Breed is stronger than pasture "; and a little plain speaking in this sense might do more than a much greater quantity of fine writing.

### Sea Water and Oysters.

The virtues of sea-water as a therapeutic agent in cases of dyspepsia and tuberculosis have been more generally recognised on the Continent than in this country. The fluid is given either in injections, or by the mouth, in small quantities before a meal, but this latter method owing to the disagreeably acrid taste of the water is not popular with patients. Carles and Laguet propose to overcome this difficulty, and at the same time to add to the efficacy of the treatment, by ordering patients to partake before meals of oysters which have previously been soaked in sea-water. The required amount of fluid has been found to be present in six large oysters. The clinical results of this treatment have been most favourable. The appetite is increased in a few days, and digestion improves. The flow of gastric juice is also increased, as shown by the examination of test meals taken before and after treatment. Clinically speaking, the oyster is a eupeptic medicament useful in many cases of ana- and hypo-chlorhydria, and in tuberculosis with dyspeptic symptoms. The good effects of the sea-water are increased by the albumen, fat, hydrocarbons, and mineral salts, especially phosphates, contained in the oyster which is also rich in glycogen. The oyster is at once a tonic and a foodstuff. Its virtues, which were recognised as early as 1827 by Sainte Marie, have recently been overlooked owing to the accusations which have been levelled against it on account of its association with typhoid bacilli, so that it is only fair, in our epoch of justice, to assist once more in the rehabilitation and revenge of the succulent mollusc whose delights are forbidden to us so long as the months, however un-summerlike in other respects, remain conventionally unsuitable to oyster-eating.

